# Predictive value of pathological and immunohistochemical parameters for axillary lymph node metastasis in breast carcinoma

**DOI:** 10.1186/1746-1596-6-18

**Published:** 2011-03-13

**Authors:** Sibel Yenidunya, Reyhan Bayrak, Hacer Haltas

**Affiliations:** 1 Department of Pathology, Fatih University Hospital, Ankara, Turkey

## Abstract

**Background/Objective:**

While several prognostic factors have been identified in breast carcinoma, the clinical outcome remains hard to predict for individual patients. Better predictive markers are needed to help guide difficult treatment decisions. Axillary lymph node metastasis (ALNM) is one of the most important prognostic determinants in breast carcinoma; however, the reasons why tumors vary in their capability to result in axillary metastasis remain unclear. Identifying breast carcinoma patients at risk for ALNM would improve treatment planning. This study aimed to identify the factors associated with ALNM in breast carcinoma, with particular emphasis on basal-like phenotype.

**Methods:**

Breast carcinoma patients (n = 210) who underwent breast conserving surgery and axillary lymph node dissection (ALND) (level I and II) or modified radical mastectomy were included in this study. Pathological and immunohistochemical data including individual receptor/gene status was collected for analysis. The basal phenotype status was ascertained using the basal cytokeratin markers CK5, CK14, CK17 and EGFR.

**Results:**

ALNM was found in 55% (n = 116) of the patients. On univariate analysis, multicentric disease, large tumor size (>2 cm), vascular and lymphatic invasion, epithelial hyperplasia, necrosis, in situ carcinoma and perineural invasion were associated with higher risk for ALNM, whereas CK5, CK14, EGFR positivity and basal-like tumor type were associated with lower risk. On multivariate analysis, CK5 positivity (OR 0.003, 95%CI 0.000-0.23, p = 0.009) and lymphatic/vascular invasion (OR 17.94, 95%CI 4.78-67.30, p < 0.001) were found to be independent predictors.

**Conclusions:**

Although the value of complete ALND has been questioned in invasive breast cancer patients, treatment decisions for breast carcinoma have been influenced by many parameters, including lymph node status. Since histopathologic characteristics and expression of biological markers varies among the same histologic subtypes of breast carcinoma, specific clinical and histopathologic features of the primary tumor and ALN status like sentinel node might be used to tailor the loco-regional and systemic treatment in different clinical settings.

## Background

Although breast cancer represents a major cause of morbidity and mortality, early detection and the use of aggressive multimodal treatment have successfully resulted in a decrease in the mortality due to this disease [1-3]. Currently, the most important prognostic factors are nodal status, tumor size, hormone receptor (HR) status, and histological grade, although numerous other clinicopathological factors and novel molecular markers have been investigated to improve the prediction of clinical outcome [4,5]. An ongoing challenge is to find improved methods of identifying and classifying groups of tumours with differing biological behaviors or responsiveness to specific therapies. Recent studies using gene expression profiling and immunohistochemistry have identified a distinct subset of breast tumours that exhibit a basal phenotype or express a gene expression signature that includes a relatively high-level expression of stratified epithelial/basal cytokeratins (CK5, CK14 and CK17) [6-12]. Triple negative (TN) breast cancers are defined as the absence of estrogen receptor (ER), progesterone receptor (PR) and HER2 expression, accounting for 10-17% of all breast carcinomas depending on the threshold used to define ER and PR positivity and the methods for HER2 assessment [13,14]. Expression of basal markers identifies a biologically and clinically distinct subgroup of TN tumors defined as basal-like breast cancer (BLBC). TN cancer and BLBC are associated with poor outcome and lack the benefit of targeted systemic therapy. The prognostic value of TN and BLBC is of paramount importance. The significance of BLBC stems not only from its poor prognostic feature but also from its distinct molecular and biological characteristics. These distinct characteristics have led to increased interest in BLBCs in an attempt to identify better systemic therapy regimens and novel therapeutic targets for these aggressive tumors. A multivariate analysis has shown the effect of the basal cell phenotype upon prognosis to be independent of other known prognostic factors, including tumour size, grade and lymph node status [8]. Several studies have shown that basal cytokeratins were associated with shorter survival in the lymph node (LN) negative tumours but not in the LN positive group [15,16]. In contrast, other studies showed an association between basal CK expression and survival in the LN positive group, but not in the LN negative group [9,17].

Axillary lymph node dissection (ALND) has been the standard of care in patients with invasive breast cancer in order to identify those with lymph node metastases, and hence estimate prognosis and guide the selection of patients for adjuvant therapy. Although no definitive evidence exists as to the therapeutic role of ALND, it is universally acknowledged that staging the axilla is mandatory for planning the proper treatment of patients with invasive breast cancer [18,19]. However, ALND has been shown to be associated with complications such as pain, lymphedema and shoulder stiffness [20]. Although it has some limitations, sentinel lymph node biopsy (SLNB) reduces the incidence of such complications and it is not associated with an increased risk of regional tumor recurrence [21]. If the axillary lymph node status could be accurately predicted prior to surgery, then selected patients who have an acceptably low probability of axillary lymph node metastasis (ALNM) might avoid a full ALND and its associated morbidity.

The purpose of this study was to estimate the likelihood of axillary lymph node involvement based on a variety of clinical and pathologic factors including basal phenotype in breast carcinoma patients and identify predictors for axillary lymph node involvement. Accurate prediction of the axillary nodal status and identifying the subgroup of basal phenotype will help optimize patient care through better planning of surgical treatment, radiotherapy and chemotherapy.

## Methods

### Patients and collection of data

This study included 210 breast carcinoma patients who underwent breast conserving surgery and ALND (level I and II) or modified radical mastectomy (MRM) in Fatih University Hospital between March 2004 and August 2010. None of the patients had distant metastasis. Medical records of the patients were reviewed to collect histopathological and immunohistochemical data including age, presence of axillary lymph node metastasis, tumor size, laterality of the tumor (unilateral or bilateral), multicentricity, estrogen receptor (ER), progesterone receptor (PR) and human epidermal growth factor receptor 2 (HER2) status. Tumour stage was determined according to the criteria established by the 7th edition of American Joint Committee on Cancer in Cancer Staging Manual [22]. Histological grade was determined according to the Elston and Ellis modification of the Scarff-Bloom-Richardson grading system [23]. The ALND specimens were examined according to local protocol where lymph nodes are identified without clearance of the fat and each node is bivalved and embedded in paraffin. At least one section of each node stained with haematoxylin and eosin (H&E) was examined, but immunohistochemistry was not used routinely to evaluate these nodal sections. For the purpose of this study, archival hematoxylin and eosin (H&E)-stained slides of cases were independently re-evaluated by three of the authors for histological typing, tumor grading, and presence of microcalcification, *in situ *carcinoma, perineural invasion, necrosis and epithelial hyperplasia. Multicentricity and multifocality were defined as the presence of tumor in multiple quadrants or the discontinuous growth of tumor in the same quadrant, respectively. An extensive intraductal component (EIC) was defined as the presence of intraductal carcinoma both in the invasive tumor and in adjacent breast tissue that comprised >25% of the tumor. In addition, representative blocks were chosen and screened for the expression of P53, EGFR, CK5, CK14, CK17 and Ki67.

### Immunohistochemical examination

Routinely processed, formalin-fixed, parafin-embedded tissue sections of 4 μm thickness were cut and mounted on polylysine-coated slides. Immunohistochemical analysis was carried out as described previously, with antibodies against ER, PR, HER2, CK5, CK14, CK17, p53, Ki67, and EGFR [24]. The primary antibodies for HER2, Ki67, CK17, EGFR, P53 and progesteron receptor were supplied by Dako Corporation (Glotsrup, Denmark). All other three markers were studied using primary antibodies supplied by Thermo Fisher Scientific (Fremont, CA, USA). The dilution factors were as follows: ER (clone SP1), 1:100; PR (PgR636), 1:50; HER2 (clone CB11), 1:200; p53 (clone DO-7), 1/50; Ki-67 (clone MIB-1), 1:150; and EGFR (clone H11), 1:50, cytokeratin 5 (clone XM26) RTU, cytokeratin 14 (clone LL002) 1:100, cytokeratin 17 (clone E3) 1:30). All primary antibodies were mouse monoclonal antibodies. Biotinylated anti-mouse antibody was used as a secondary antibody and streptavidin horseradish peroxidase (Zymed laboratories, San Francisco, CA) methods were used according to the instructions provided by the manufacturer. Slides were counterstained with Harris hematoxylin. Positive and negative controls for each marker were used according to the supplier's data sheet.

Immunohistochemical results were assessed by at least two pathologists and scored semiquantitatively. ER and PR positivity was defined as the presence of 10% or more positively stained nuclei in ten high-power fields [25]. A positive HER2 was recorded only if immunostaining was seen 3+ strong membranous staining [26,27]. Positivity for CK5, CK14 and CK17 was defined as the detection of at least 1% of invasive tumor cells showing strong cytoplasmic and membranous staining [9,27]. Immunostaining for EGFR was interpreted as positive when at least 10% of tumor cells showed positive strong membranous staining. P53 immunostaining was interpreted as positive when more than 10% of tumor cells exhibited strong nuclear staining, and Ki-67 expression was considered low when <20% of cells were stained. Tumors were classified as follows: luminal A (ER+ or PR+ and HER2-), luminal B (ER+ or PR+ and HER2+), HER2 (ER- and PR- and HER2+). A tumor was defined as triple-negative when it is negative for all three of the ER, PR receptors and HER2 and a triple-negative tumor is defined as basal-like when it is positive for at least one of CK5, CK14, CK17 or EGFR.

### Statistical analysis

Statistical Package for Social Sciences (SPSS) version 15 was used for the analysis of data. Categorical data are presented as frequency (percentage) and continuous data are presented as mean ± standard deviation. For the comparison of categorical data, Chi-square test or Fisher's exact test was done, and continuous variables were compared using unpaired t-test. In order to identify the independent predictors of axillary lymph node metastasis, logistic regression was used. Odds ratios of the significant predictors are provided along with 95% confidence intervals (CI). Patients with missing data were excluded from corresponding analyses. A p value < 0.05 was considered as an indication of statistical significance.

## Results

Patient and tumor characteristics stratified by ALNM status are presented in Table 1. The mean age of the patients was 53.5 ± 13.0 y (median: 52 y, range: 19-85 y). Fourteen patients were below age 35 y, 75 between 35-50 y and 121 patients were older than 50 y. Distribution of the tumors with regard to size was as follows: 9 (4.3%), pT1a; 23 (11%), pT1b; 54 (26%), pT1c; 99 (47%), pT2; 13 (6%), pT3; and 10 (4.7%) pT4. Axillary lymph node metastasis was found in 55% (n = 116) of the study group. Data on sentinel lymph node were available in 31.5% (n = 66) of patients and 14.8% (n = 31) was positive. In the remaining patients, sentinel lymph node was not specifically assessed, although lymph node dissection was performed. The sentinel lymph node status correctly predicted the status of the axilla in 97% of the cases (64/66). The sentinel-node status was positive in 31 cases (47%), and negative in the remaining 35 cases (53%). One of the 35 cases with a negative sentinel-node status had non-sentinel node involvement. Among the 144 patients in whom sentinel node status was not assessed, 60 (42%) did not have axillary lymph node involvement and remaining 84 cases (58%) had ALNM. Patients with or without SLN assessment did not differ with regard to the frequency of ALNM: 42% (32/66) versus 58% (84/44), p = 0.182. The mean number of reactive lymph nodes was 17.2 ± 9.5 (median: 17, range: 0-46) in the whole study group and the mean number of metastatic lymph nodes was 6.0 ± 6.8 (median: 4, range: 0-39) in patients with ALNM.

**Table 1:** Patient and tumor characteristics (n = 210)

Factor		Node-negative Node-positive		P value
	# of sample	(n = 94)	(n = 116)	
**Age (yr)**	**210**	**55.8 ± 12.4**	**51.7 ± 13.3**	**0.020**
**Age groups (yr)**				
≤49	79	31 (33.0%)	48 (41.4%)	0.211
≥50	131	63 (67.0%)	68 (58.6%)	
**Tumor size (mm)**				
Tmicro	3	3 (3.2%)	0	0.001
T1a	6	3 (3.2%)	3 (2.6%)	
T1b	23	13 (14.0%)	10 (8.7%)	
T1c	54	33 (35.5%)	21 (18.3%)	
T2	99	37 (39.8%)	62 (53.9%)	
T3	13	4 (4.3%)	9 (7.8%)	
T4	10	0	10 (8.7%	
**Histologic grade**				
1	43	19 (20.2%)	24 (20.7%)	0.091
2	69	37 (39.4%)	32 (27.6%)	
3	86	31 (33.0%)	55 (47.4%)	
Unknown	12	7 (7.4%)	5 (4.3%)	
**Lymphovascular invasion**				
Negative	100	75 (79.8%)	25 (21.6%)	<0.001
Positive	105	16 (17.0%)	89 (76.7%)	
Unknown	5	3 (3.2%)	2 (1.7%)	
**Perineural invasion**				
Negative	133	69 (73.4%)	64 (55.2%)	0.003
Positive	72	22 (23.4%)	50 (43.1%)	
Unknown	5	3 (3.2%)	2 (1.7%)	
**Multicentricity**				
Negative	153	75 (79.8%)	78 (67.2%)	0.300
Positive	56	18 (19.1%)	38 (32.8%)	
Unknown	1	1 (1.1%)	0	
**In situ carcinoma**				
Negative	46	29 (30.9%)	17 (14.7%)	0.005
Positive	159	63 (67.0%)	96 (82.8%)	
Unknown	5	2 (2.1%)	3 (2.5%)	
**Microcalcification**				
Negative	136	66 (70.2%)	70 (60.3%)	0.087
Positive	67	24 (25.5%)	43 (37.1%)	
Unknown	7	4 (4.3%)	3 (2.6%)	
**Epithelial hyperplasia**				
Negative	104	57 (60.6%)	47 (40.5%)	0.002
Positive	96	32 (34.0%)	64 (55.2%)	
Unknown	10	5 (5.4%)	5 (4.3%)	

Univariate analysis for relationship between the clinical and pathological variables and the ALNM status is presented in Table 2. Age, tumor size, multicentric disease, lymphovascular invasion, epithelial hyperplasia, necrosis, in situ carcinoma and perineural invasion were found to be significantly associated with ALNM. The mean age of the patients with ALNM was significantly lower indicating an increased risk for young patients. Multicentric disease, large tumor size (>2 cm), lymphovascular invasion, epithelial hyperplasia, necrosis, extensive in situ carcinoma and perineural invasion were significantly more common among patients positive for ALNM; thus associated with higher risk. Patients with ALNM had similar distribution pattern for histological type (p = 0.169) and tumor grade (p = 0.091). Poorer histologic grade was associated with ALNM but the relationship was not significant.

**Table 2:** Univariate analyses of factors for their association with ALNM

Factor	Unadjusted odds ratio and 95% confidence interval	ALNM negative (n = 94)	ALNM positive (n = 116)	P value
Age (yr), mean ± SD	-	55.8 ± 12.4	51.7 ± 13.3	0.020

Age groups (yr)				
<50	0.70 (0.40-1.23)	31/94 (33.0%)	48/116 (41.4%)	
≥50		63/94 (67.0%)	68/116 (58.6%)	02.12

Tumor size (mm)				
T1a (0-5.0)	Reference	6/93 (6.5%)	3/115 (2.6%)	0.001
T1b (5.1-10)	1.54 (0.31-7.72)	13/93 (14.0%)	10/115 (8.7%)	
T1c (10.1-20.0)	1.27 (0.29-5.65)	33/93 (35.5%)	21/115 (18.3%)	
T2 (20.1-50.0)	3.35 (0.79-14.21)	37/93 (39.8%)	62/115 (53.9%)	
T3-T4 > 50	9.50 (1.65-55.0)	4/93 (4.3%)	19/115 (16.5%)	

Tumor size >20 mm	3.02 (1.70-5.36)	41/93 (44.1%)	81/115 (70.4%)	<0.001

Histologic grade				
1	Reference	19/87 (21.8%)	24/111 (21.6%)	
2	0.68 (0.32-1.47)	37/87 (42.5%)	32/111 (28.8%)	0.332
3	1.40 (0.67-2.96)	31/87 (35.6%)	55/111 (49.5%	0.371

Bilateral disease	1.03 (0.37-2.88)	7/92 (7.6%)	9/115 (7.8%)	0.954

Multicentric disease	2.03 (1.07-3.87)	18/93 (19.4%)	38/116 (32.8%)	0.030

Lymphovascular invasion	16.69 (8.30-33.56)	16/91 (17.6%)	89/114 (78.1%)	<0.001

Microcalcification	1.69 (0.93-3.08)	24/90 (26.7%)	43/113 (38.1%)	0.087

Epithelial hyperplasia	2.43 (1.37-4.31)	32/89 (36.0%)	64/111 (57.7%)	0.002

Necrosis	2.12 (1.10-4.08)	17/89 (19.1%)	38/114 (33.3%)	0.024

In situ carcinoma	2.60 (1.32-5.12)	63/92 (68.5%)	96/113 (85.0%)	0.005

Perineural invasion	3.02 (1.70-5.36)	22/91 (24.2%)	50/114 (43.9%)	0.003

Unless otherwise stated, data are expressed as n (%). SD, standard deviation; ALNM, axillary lymph node metastasis

Table 3 shows the univariate analysis results for immunohistochemical findings. A positive HER2 status was not related to positive ALN status. In addition, ER and PR expression did not predict the ALN status. The distribution of tumors by ER/PR/HER2 status was similar among the groups with and without ALNM (p = 0.781). To investigate the relationship between molecular subtype and ALNM status, we used immunohistochemical markers that have been previously compared with gene expression profiles as surrogates and presenting characteristics of breast carcinoma [28]. Although triple negative subgroup was not associated with increased risk for ALNM, basal phenotype showed a stronger association with negative ALN tumors (75%).

**Table 3:** Univariate analysis of immunohistochemical findings and their association with axillary lymph node metastasis

Factor	Unadjusted odd ratio and 95% confidence interval	ALNM negative (n = 94)	ALNM positive (n = 116)	P value
ER	1.14 (0.62-2.07)	63/92 (68.5%)	79/111 (71.2%)	0.677

PR	1.13 (0.62-2.07)	64/92 (69.6%)	80/111 (72.1%)	0.695

HER2	1.28 (0.64-2.56)	17/92 (18.5%)	25/111 (22.5%)	0.479

CK5	0.25 (0.10-0.61)	21/86 (24.4%)	7/95 (7.4%)	0.002

CK14	0.28 (0.09-0.91)	12/73 (16.4%)	4/77 (5.2%)	0.034

CK17	0.38 (0.09-1.52)	7/72 (9.7%)	3/77 (3.9%)	0.198

P53	0.95 (0.48-1.88)	23/71 (32.4%)	25/80 (31.3%)	0.880

Ki67	1.51 (0.80-2.82)	33/78 (42.3)	42/80 (52.5%)	0.200

EGFR	0.35 (0.15-0.82)	20/61 (32.8%)	10/69 (14.5%)	0.013

***ER/PR/HER2 profile***				

ER/PR+, HER2-	Reference	59/92 (64.1%)	71/111 (64.0%)	

ER/PR+, HER2+	1.50 (0.48-4.70)	5/92 (5.4%)	9/111 (8.1%)	0.488

ER/PR-, HER2+	1.11 (0.49-2.53)	12/92 (13.0%)	16/111 (14.4%)	0.806

ER/PR-, HER2-	0.78 (0.36-1.71)	16/92 (17.4%)	15/111 (13.5%)	0.532

Triple-negative tumor	0.74 (0.35-1.60)	16/92 (17.4%)	15/111 (13.5%)	0.445

Basal-like tumor	0.25 (0.08-0.80)	12/92 (13.0%)	4/111 (3.6%)	0.017

Unless otherwise stated, data are expressed as n (%).ALNM, axillary lymph node metastasis; ER, estrogen receptor; PR, progesterone receptor; HER2, human epidermal growth factor receptor 2; CK, cytokeratin

Among individual immunohistochemical parameters, CK5, CK14, and EGFR positivity was associated with lower risk for ALNM, whereas groups did not differ with regard to the positivity of other individual molecular parameters. The frequencies of triple negative tumors were also similar in the two groups; however, basal-like tumors were more frequent among patients with ALNM negative.

An additional multivariate logistic regression analysis was conducted including the variables that were found to have a significant association in univariate analysis and their associations with ALNM status are presented in Table 4. Only CK5 positivity, lymphovascular invasion and age were emerged as independent predictors for ALNM. CK5 positivity was associated with a lower risk (adjusted OR 0.003, 95%CI 0.000-0.23, p < 0.009) and lymphatic/vascular invasion (LVI) was associated with a higher risk (adjusted OR 17.94, 95%CI 4.78-67.30, p < 0.001) for ALNM. Although marginal, age also emerged as a significant predictor, with increasing age associated with a lower risk (adjusted OR 0.95, 95%CI 0.91-1.00, p < 0.049).

**Table 4:** Multivariate analysis of factors with significant association with axillary lymph node metastases in univariate analysis

Factor	Adjusted odds ratio (95% confidence interval)	P value
Age	0.95 (0.91-1.00)	0.049

Multicentric disease	1.46 (0.41-5.22)	0.561

Tumor size >2 cm	2.48 (0.81-7.59)	0.113

Vascular and lymphatic invasion	17.94 (4.78-67.30)	<0.001

Epithelial hyperplasia	1.73 (0.52-5.77)	0.373

Necrosis	0.49 (0.13-1.84)	0.292

In situ carcinoma	1.70 (0.42-6.84)	0.455

Perineural invasion	1.13 (0.31-4.20)	0.853

CK5 positivity	0.003 (0.000-0.23)	0.009

CK14 positivity	5.22 (0.19-140.76)	0.326

EGFR positivity	3.94 (0.55-28.17)	0.171

Basal-like tumor	3.85 (0.18-83.02)	0.389

When patients having the two significantly favorable parameters were specifically analyzed, i.e, patients with CK5 expression and without lymphovascular invasion, 15 patients were identified. None of these patients had ALNM.

## Discussion

A common first route of spread for breast carcinoma is through the axillary lymph nodes, and the incidence of ALNM increases with larger tumors. Nodal status is the most powerful independent prognostic factor in breast cancer and remains the most important feature for defining risk category. There is evidence that overall survival decreases as the number of positive node increases [4,29]. According to St. Gallen experts, involvement of four or more nodes in the axilla by itself indicated high-risk, but patients with one to three nodes involved required HER2/neu overexpression or amplification to be included in the high-risk group, with other patients with one to three nodes included in the intermediate-risk category [30]. Attempts have been made to identify factors that may predict an increase risk of nodal involvement in this group of patients. Although nodal micrometastases were prognostically relevant in several studies [31,32], neither they nor isolated tumor cells in lymph nodes are considered in risk allocation. Increasing size of the tumor has been found to be predictive of ALNM. Even patients with T1a and T1b disease have significant nodal involvement (5-15%) [33-36]. This study found a 29% overall incidence of ALNM in patients with tumors ≤20 mm. There was no difference in the distribution of ALNM frequency among the different histologic types.

Multicentricity has been evaluated as a potential predictive factor of ALNM. When a combined diameter assessment was used, the frequency of lymph node positivity was not significantly different in multifocal versus unifocal cases [37,38]. However, multicentric and multifocal breast cancer is associated with increased nodal involvement compared to similar unifocal disease and the tendency of breast tumors to metastasize is a reflection of the total tumor load [38]. Classification of patients with breast cancer based on the size of the dominant lesion, without taking into account multicentricity, may not accurately reflect the risk of ALNM in patients with small, screen-detected cancers. This may, in turn lead to over- or under-treatment of some of these patients. Univariate analysis of this study identified multicentricity as a significant factor for ALNM. The number of foci should be considered as an independent prognostic parameter, which is currently not reflected in the TNM classification. Therefore, cases with multicentric tumours should be analyzed separately and should be included in the risk assessment by re-evaluating the current TNM classification.

In univariate analysis, there was a positive relation between nodal involvement and EIC. It is possible that EIC sometimes was associated with unrecognized multifocality of infiltrating cancer predisposing to nodal spread. Further research is required to explore this and other possible explanations.

Lymphovascular invasion has been proved to be the strongest independent predictor of nodal involvement in two series, and the grading system for lymph-vessel tumor embolus is a very useful histological grading system for accurately predicting lymph node metastasis by IDCs [39,40]. In this study, both univariate and multivariate analysis identified lymphovascular invasion as significant predictors for ALN involvement.

Despite controversial data available on the value of age as a prognostic factor, the prognosis of breast cancer in very young women is generally considered to be unfavorable. Age has been found to be an independent prognostic factor in the multivariate analysis with women aged 35 years or younger having a shorter loco-regional recurrence-free distant relapse and shorter overall survival. When the data was matched for stage and lymph node status, patients ≤35 years of age continued to show a poorer 10-year distant relapse free survival [41]. Ten-year disease free survival (DFS) and overall survival were worse in younger than in older (≥35 years) patients. Of interest, younger patients with ER positive tumors had a poorer DFS than patients with ER negative tumors. In contrast, among older patients the DFS was similar irrespective of ER status. Saghir et al. showed that young age had a negative impact on the survival of patients with positive axillary lymph nodes and positive hormonal receptors [42]. According to Colleoni and colleagues, compared with less young, very young patients with endocrine responsive and node-negative breast cancer have a worse prognosis [43]. Young patients tend to have larger tumor sizes, more positive lymph nodes, more negative hormone receptors, higher tumor grades than their older counterparts [4,11,14,18,26,29]. The issue remains controversial and not all studies reported age as an adverse prognostic factor [18-21,26,27,29]. In line with these findings, although the significance is marginal, this study identified young age as a significant predictor of ALNM. This negative predictive effect of age on nodal involvement is consistent with earlier evidence and indicates that breast tumours can be more aggressive in younger women [44-46]. Alternatively, this may be due to the longer period of exposure to the screening service of older women, when compared to the younger women; thus reflecting a cumulative protective effect of repeated screening.

The potential poor survival or early recurrence associated with CK5/6 and CK17 expression in tumor cells was first reported by Dairkee *et al. *in 1987 [47]. Although IHC-based assays do not provide so much biological insight into tumor biology as mRNA-based assays that include thousands of genes, this IHC assay allowed classification of tumors into categories based on the associations between intrinsic subtypes and proliferation rates, overall survival, *TP53 *status, and *BRCA1 *mutation status [8-10,15,48]. In order to facilitate the investigation of the ALNM frequencies in basal-like breast cancer subtype, this study used a refined an IHC-based assay. Basal markers are not routinely used in the standard histological diagnosis of breast cancer. As existing prognostic markers do not identify this group, patients with basal-like and non-basal-like tumours are currently treated similarly. In this study, when all tumors were classified into five groups based on ER/PR/HER2 and basal-cytokeratin expressions, ALNM rate was lower in only basal-like phenotype. In addition, this study identified lack of CK5 expression as an independent predictor of ALNM, with tumors expressing CK5 bearing a significantly smaller risk (adjusted OR = 0.003, 95% CI 0.000-0.23, p = 0.009).

Marked differences in the extent of lymph node involvement, multicentric/multifocal disease, lymphovascular invasion (LVI), and extensive intraductal component were observed among subtypes. Only the basal subtype was found to differ significantly from the luminal A subtype with regard to the risk of nodal metastasis. This subtype was a significant predictor of having involvement of four or more nodes on multivariate analysis. However, some studies have shown in patients without ALNM that expression of basal CK was associated with a poor prognosis [15,49]. New prognostic markers are quite important for this group of patients since the prognosis for node-negative patients is less clear, and the clinical decision to give or withhold systemic therapy is difficult, depending only on tumor size and grade. CK5 expression appears to be a useful marker to define the group of breast tumors with a poor prognosis even in the absence of ALNM.

Mutations in the tumor suppressor gene, p53, are present in 18-25% of primary breast carcinomas [50,51]. A previous study found significant association between anti-p53 antibodies and tumor size, histological grade, and the number of axillary lymph nodes involved [52]. TN breast cancers more frequently show p53 nuclear expression; therefore, are likely to harbour *TP53 *gene mutations more frequently [53]. In this study, patient with P53 expression were present in 49% HER2 subtype, and in 41% basal-like phenotype. However, p53 expression did not emerge as a significant factor associated with ALNM. Expression of p53 could provide information concerning a poor outcome in triple-negative breast cancer. There is no definite answer to optimal management of triple-negative tumors at this moment. In such cases, consideration might well be given to more aggressive or alternative treatment.

Proliferative activity of tumour cells assessed by immunohistochemical Ki-67 expression is one of several prognostic indicators in breast cancer. Intriguingly, all the studies [54-56] have shown a statistical correlation with clinical outcome irrespective of cut-off points. Previous studies have reported significant associations between high Ki-67 index and lymph node status [57]. In contrast, similar to the findings of this study, a recent study have shown that Ki-67 did not appear to be a helpful predictor [58].

In this study, univariate analysis identified significant associations between ALNM and twelve factors: age, multicentric disease, tumor size, vascular and lymphatic invasion, epithelial hyperplasia, necrosis, in situ carcinoma, perineural invasion, basal-like phenotype, and CK5, CK14 and EGFR expressions. However, only age, CK5 expression and lymphatic/vascular invasion remained to be significant predictors on multivariate analysis. Increasing age and CK5 expression was associated with decreased risk whereas lymphatic and vascular invasion was associated with an increased risk.

Clinical assessment of the axilla, tumor palpability and the method of detection were not uniformly documented in our data set, thus were not included in the analysis. This represents a limitation of this study. Another potential weakness of this report may be that ALN status we based on the results from a mixture of complete axillary node dissection and sentinel lymph node procedure. However, it has previously been shown that predictors for ALN status are independent of how the lymph node resection was performed although the metastatic detection rate in lymph nodes may be higher using the sentinel node procedure since higher number of metastases has been detected thorough histological examination of the sentinel lymph node biopsies [59]. In general, patients can be selected for breast-conserving surgery with a high degree of accuracy by history, physical examination, and diagnostic mammography [60]. This study identified age, CK5 expression and vascular/lymphatic invasion as three variables associated with high-risk for ALNM. The latter two can be identified preoperatively with reasonable accuracy by pathologic evaluation of core needle biopsies [61]. As a result, pathologists can accurately assess the true malignant potential of IDCs by seeking lymph-vessel tumor emboli as part of a histological prognostic classification. In addition, patients with basal-like carcinomas may be less sensitive to standard adjuvant chemotherapy than with other types; necessitating novel therapeutic approaches.

## Conclusion

In conclusion, findings of this study suggest that the inclusion of biomarker profiles to predict nodal status at diagnosis may provide additional information that has the potential to be useful for decisions regarding breast-conserving therapy, axillary surgery, and locoregional radiation.

## List of a abbreviation

ALNM: Axillary lymph node metastasis; ALND: Axillary lymph node dissection; ALN: Axillary lymph node; CK: Cytokeratin; IHC: Immunhistochemistry; HR: Hormone Receptor; TN: Triple Negative; ER: Estrogen Receptor; PR: Progesterone Receptor; BLBC: Basal-like Breast Cancer; LN: Lymph Node; SLNB: Sentinel Lymph Node Biopsy; CI: Confidence Intervals; EIC: Extensive Intraductal Component; IDC: Invasive Ductal Carcinoma; LVI: Lymphovascular Invasion; DFS: Disease Free Survival.

## Competing interests

The authors declare that they have no competing interests.

## Authors' contributions

SY carried out the data collection, the pathological and IHC evaluation and interpretation, drafting and wrote the final manuscript; RB and HH participated in pathological and IHC evaluation and interpretation. All authors read and approved the final manuscript.
